# Identification of Novel Single Nucleotide Polymorphisms in Inflammatory Genes as Risk Factors Associated with Trachomatous Trichiasis

**DOI:** 10.1371/journal.pone.0003600

**Published:** 2008-10-31

**Authors:** Berna Atik, Troy A. Skwor, Ram Prasad Kandel, Bassant Sharma, Him Kant Adhikari, Lori Steiner, Henry Erlich, Deborah Dean

**Affiliations:** 1 Center for Immunobiology and Vaccine Development, Children's Hospital Oakland Research Institute, Oakland, California, United States of America; 2 Lumbini Rana-Ambika Eye Hospital, Bhairahawa, Nepal; 3 Roche Molecular Systems, Pleasanton, California, United States of America; 4 HLA Laboratory, Children's Hospital Oakland Research Institute, Oakland, California, United States of America; 5 Department of Medicine, University of California at San Francisco School of Medicine, San Francisco, California, United States of America; 6 Joint Graduate Group in Bioengineering, University of California at San Francisco, San Francisco, California, United States of America; 7 University of California Berkeley, Berkeley, California, United States of America; Ohio State University Medical Center, United States of America

## Abstract

**Background:**

Trachoma is the leading preventable cause of global blindness. A balanced Th1/Th2/Th3 immune response is critical for resolving *Chlamydia trachomatis* infection, the primary cause of trachoma. Despite control programs that include mass antibiotic treatment, reinfection and recurrence of trachoma are common after treatment cessation. Furthermore, a subset of infected individuals develop inflammation and are at greater risk for developing the severe sequela of trachoma known as trachomatous trichiasis (TT). While there are a number of environmental and behavioral risk factors for trachoma, genetic factors that influence inflammation and TT risk remain ill defined.

**Methodology/Findings:**

We identified single nucleotide polymorphisms (SNP) in 36 candidate inflammatory genes and interactions among these SNPs that likely play a role in the overall risk for TT. We conducted a case control study of 538 individuals of Tharu ethnicity residing in an endemic region of Nepal. Trachoma was graded according to World Health Organization guidelines. A linear array was used to genotype 51 biallelic SNPs in the 36 genes. Analyses were performed using logic regression modeling, which controls for multiple comparisons. We present, to our knowledge, the first significant association of TNFA (-308GA), LTA (252A), VCAM1 (-1594TC), and IL9 (T113M) polymorphisms, synergistic SNPs and risk of TT. TT risk decreased 5 times [odds ratio = 0.2 (95% confidence interval 0.11.–0.33), *p* = 0.001] with the combination of TNFA (-308A), LTA (252A), VCAM1 (-1594C), SCYA 11 (23T) minor allele, and the combination of TNFA (-308A), IL9 (113M), IL1B (5′UTR-T), and VCAM1 (-1594C). However, TT risk increased 13.5 times [odds ratio = 13.5 (95% confidence interval 3.3–22), p = 0.001] with the combination of TNFA (-308G), VDR (intron G), IL4R (50V), and ICAM1 (56M) minor allele.

**Conclusions:**

Evaluating genetic risk factors for trachoma will advance our understanding of disease pathogenesis, and should be considered in the context of designing global control programs.

## Introduction

Trachoma is a chronic ocular disease caused by *Chlamydia trachomatis*, although other chlamydial species have recently been implicated in trachomatous inflammation and disease [1]. Trachoma remains the primary preventable cause of visual impairment and blindness in the world [2]. Repeated and/or persistent ocular infections and inflammation can result in subsequent conjunctival scarring and fibrosis, leading to entropion (distorted eyelid) and trichiasis (TT;≥one in-turned eyelash touching the globe of the eye) [3]. TT represents an important threat to vision because of the development of corneal abrasions and subsequent opacity [3], [4].

The World Health Organization (WHO) developed the SAFE strategy [Surgery, Antibiotic treatment, Facial cleanliness, Environmental improvement] to eliminate blinding trachoma by the year 202 [5]. However, mass or targeted antibiotics has resulted in the recurrence of trachoma and infection after treatment cessation in multiple studies [6]–[9]. Consequently, there has been a continued focus on understanding host immune responses for each grade of trachomatous disease along with chlamydial infection to provide the knowledge to enhance control programs and vaccine development, and assess vaccine efficacy once one becomes available.

Humoral and cell mediated immune responses are considered essential in both the clearance of chlamydial infection and in the immunopathogenesis of trachomatous disease, although the pathogenic mechanisms remain unclear. Cell mediated immunity is primarily controlled by cytokines at the local mucosal microenvironment [10]. The contribution and presence of a particular cytokine may vary according to the stage of infection or grade of trachoma. Some studies have evaluated cytokine mRNA gene expression in trachoma and found that transforming growth factor beta (TGF-β) and the pro-inflammatory cytokines, interleukin-1 beta (IL-1β) and tumor necrosis factor alpha (TNF-α), were significantly elevated in trachomatous follicular inflammation (TF) and trachomatous scarring (TS) [11]–[13]. We recently expanded on the characterization of cytokines and also identified chemokines involved in the immunopathology of different grades of trachoma using a more robust approach that involved conjunctival mucosal protein quantitation. Our findings highlight the importance of Th1 cytokines in protection against TS as evidenced by decreased protein levels for IL-12p40 compared with controls [14]. Additionally, we identified involvement of the Th2 cytokines IL-4 and IL-13 in eliciting protective immunity, and IL-10 and IL-15 as possible Th3/Tr1 cytokines and risk factors for TS. IL-1β was also a strong risk factor for scarring while its antagonist, IL-1Ra, was protective. *C. trachomatis* infection was significantly associated with elevated proinflammatory TNF-α and IL-6 cytokines, the Th3/Tr1 IL-10 and IL-15 cytokines, and the Th1-associated chemokines MIP-1β and RANTES in the presence of trachomatous disease, suggesting an added microbial influence on trachoma pathogenesis.

To further characterize the host immune response, it will be necessary to evaluate host genetic susceptibility to the development of trichiasis. This is particularly important because some individuals in trachoma endemic areas develop severe inflammation with each infection while others in the same community do not, despite similar environmental and ethnic backgrounds [3]. Furthermore, the former are significantly more likely to develop TS and TT [3], [15].

There have been some studies evaluating the association between single nucleotide polymorphisms (SNPs) among inflammatory genes and trachomatous disease [16]–[20]. In Gambia, Th1/Th2/Th3 gene SNPs, including IFNG (+3234 *C*), IL-10 (+5009 *G*, −1082 *G* only in the Mandinka ethnic group, and −3575*A*), and TNFA (−308 *A*), were found to be associated with a higher odds of having TS [16], while TNFA (−308 *GA*) and lymphotoxin alpha [LTA, +252 *GG*, and inhibitor of kappa light chain gene enhancer in B-cells-like (IKBL, −63 *TT*) SNPs were significantly associated with TS or TT [16], [17], [19], [20]. SNPs in LTA (+77 *G>T*), TNFA (−238 *G>A*, −376 *G>A*), inhibitor of kappa light chain gene enhancer in B-cells, alpha (IKBA, −881 *A>G*, −826 *C>T*, −297 *C>T*), IFNG (−1616 *C>T*, +2200 *T>C*, +5612 *C>T*), IL-4 (−590 *T>C*) and IL10 (−1082 *T>C or A>G*, −819 *C>T*, −592 *A>C* or *G>T*) were not associated with TS or TT [16]–[20]. For the IL10 (−1082) polymorphism, the literature is contradictory (T>C [19] or A>G [17]). More recently, researchers found that heterozygosity for the Q279R polymorphism in matrix metalloproteinase 9 (MMP-9), which plays a role in tissue remodeling, was associated with lower risk for scarring trachoma compared to homozygosity for the “*A*” allele (i.e.Q279) [21]. These findings suggest a role for SNPs in the pathogenesis of trachoma. However, all of these studies were conducted in Gambia, and only two of these used TT as the outcome. Similar studies need to be replicated among trachoma endemic populations in other countries. Additionally, there are a plethora of inflammatory genes and SNPs that remain to be characterized in association with trachoma.

The purpose of the present study was to expand on previous SNP research and determine whether subjects with TT were more likely to have particular SNPs compared to subjects without any evidence for trachoma. We screened 51 SNPs derived from 36 biologically plausible candidate genes encoding proteins in inflammatory pathways to identify genetic markers that may assist in elucidating the pathogenesis of trachoma in a Nepali population. To our knowledge, this is the first study to examine such a large array of inflammatory SNP markers in association with trachomatous disease.

## Materials and Methods

### Study Population and Trachoma Grading

The study and written informed consent for the study were approved by the Institutional Review Boards of Children's Hospital and Research Center at Oakland, CA, and the Nepal Netra Jhoti Sang, Kathmandu, Nepal, according to the Convention of Helsinki. A survey was conducted in a trachoma endemic region of Kapilvastu District, Lumbini Zone, Nepal. All study participants were of the Tharu ethnic group. Trachoma grading was performed using the modified grading scale according to WHO guidelines as described previously [22], [23]. Briefly, subjects with <5 follicles on the lower 2/3 of the upper tarsus were defined as having no trachoma. Trachomatous inflammation, follicular (TF) was defined as five or more follicles in the upper tarsus, trachomatous inflammation, intense (TI) as intense inflammatory thickening of upper tarsal conjunctiva with indistinct deep tarsal vessels, trachomatous scarring (TS) as the presence of scarring in the upper tarsus, and trachomatous trichiasis (TT) as at least one in-turned eyelash rubbing the eyeball or history of epilation (removal of in-turned eyelashes). After obtaining informed consent, research staff enumerated and clinically examined all household members in two villages in the district.

Whole blood samples collected in ethylenediaminetetraacetic acid (EDTA) containing tubes and conjunctival swabs were obtained from all villagers as previously described [1], [23]. Briefly, the upper tarsal conjunctivae of both eyes were swabbed with a Dacron swab (American Scientific Products, McGraw Park, IL). Swabs were placed in collection media (M4-RT; Micro-Test, Lilburn, GA). A new set of gloves was used for each subject to prevent cross-contamination of *C. trachomatis*. All the samples were dated and coded with a unique ID number to maintain confidentiality and to process samples in a masked fashion. The samples were immediately placed in liquid nitrogen transport tanks for transfer to Children's Hospital Oakland Research Institute where the samples were then stored at −80°C until processed.

### Detection of Chlamydia trachomatis

The Amplicor-PCR assay (Roche Diagnostics, Branchburg, NJ) was used to detect *C. trachomatis* for each conjunctival sample according to the manufacturer's instructions and as we described previously [1], [14], [23]. Of note is that gloves were used in between subjects to prevent cross-contamination of the collected samples. Optical density (OD) was measured at 450 nm using an automated microwell plate reader. Samples were defined as positive with an OD_450 nm_ of ≥0.8, negative with an OD_450 nm_ of ≤0.2, and equivocal with an OD_450 nm_ between 0.2 to 0.8. All equivocal samples were reevaluated by an in-house validation PCR test to further assess the presence or absence of chlamydiae as we have described [1], [23]. Briefly, DNA isolated from conjunctival swabs was amplified using primers that flank the *ompA* gene. PCR products of the same molecular weight size as the PCR positive control were considered positive as long as the negative control was negative.

### Extraction of Genomic DNA

Genomic DNA was extracted from whole blood using Genovision GenoM™ (GenoVision Inc., West Chester, PA), according to manufacturer's instructions. The principle of DNA isolation and purification by GenoVision technology is that DNA binds to the glass surface of magnetic beads in the presence of a chaotropic solution. Cell lysis is followed by binding of DNA to magnetic beads, washing and elution of the DNA. The concentration of the DNA was determined using a spectrophotometer at OD_260 nm_.

### Linear array for SNP detection

**Figure 1:**
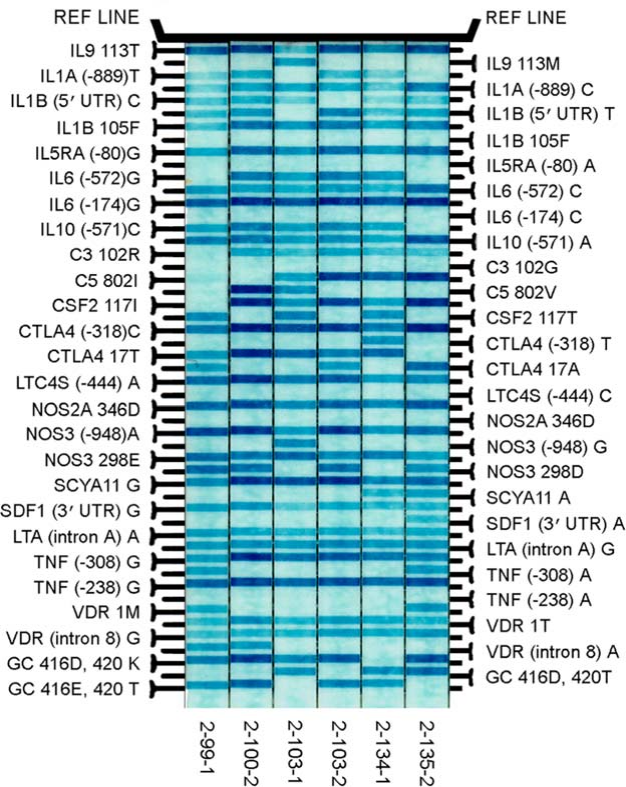
Representative linear array results for Strip 1 for samples from six Tharu individuals from a trachoma endemic area of Nepal. Bands represent wild type and SNP probes (see methods). Strip 1 contains 25 SNPs. Strip 2 contains 26 SNPs (not shown). To the left and right of the probes are the names of the alleles; horizontal blur lines represent amplified alleles.

We used an immobilized probe linear array assay developed by Roche Molecular Systems (Alameda, CA) to genotype 51 biallelic SNPs in 36 genes associated with inflammation as previously described by Barcellos et al.[24], although in the referenced study, only 34 genes were used. The selection of SNPs was based on publicly available databases that included Caucasian and Chinese data. Briefly, 25 ng of genomic DNA from each sample was amplified by multiplex PCR using biotinylated primers for all 51-gene polymorphisms. Two probes were designed for each biallelic site to detect and distinguish between variant sequences. The PCR product was denaturated with 10 µl Amplicor base streptavidin-HRP and a colorless soluble substrate, which is converted into a blue precipitate. Bands on the developed arrays were aligned to a guide to identify the respective allele, and the arrays were scanned into a computer (Figure 1). SNP interpretations were made independently by two individuals masked as to all subject data.

### Data Analysis

Allele and genotype frequencies were compared for cases (TT) vs. controls (no evidence for TF, TI, TS or TT) by chi-square test. Hardy-Weinberg equilibrium (HWE) was tested for each SNP. We performed simple and multiple logistic regression analysis; age, sex, having TF, TI, TS, TT and *C. trachomatis* infection status were covariates included in multiple logistic regression. Finally, we performed logic regression to analyze interactions among multiple SNPs.

Logic regression was used to identify gene-gene interactions [25]. Correction for multiple comparisons is not considered necessary in logic regression since the multi-step model selection is very precise and thereby accounts for multiple comparisons [25]. Instead of cross-product terms that are used in logistic regression, logic builds Boolean expressions in the form of “and/or” trees (L) to identify gene interactions. A simulated annealing algorithm is used to predict logic trees. The annealing algorithm works by starting from a particular state and then selecting a move to a new state. The score of the new state is compared with the score of the old state. If the score of the new state is better than the score of the old state, the move is accepted. We chose to use the simulated annealing algorithm because it is a robust technique for nonlinear models even though it is computationally very demanding.

We used logic regression within the context of logistic regression. In this study, the outcome was binary [subjects having TT (cases) or no evidence for any trachoma grade (controls)]. Deviance was used as the scoring function. We used the 10-fold cross-validation test, comparing the best model to models of different sizes. In addition, we used randomization tests to determine optimal model size. In order to carry out a null-model test for signal in the data, an initial “best scoring” model, allowing up to two trees and eight leaves (the default maximum tree size provided by the function “logreg”) was obtained. The score of this “best model” was then compared to the distribution of scores obtained by repeatedly permuting the outcome and refitting the data. The proportion of scores from the permuted data that are better than the best score of the original data is reported as an exact p-value. We used the more conservative p-value of less than 0.001 as a cut-off for statistical significance in the logic analysis.

## Results

### Clinical and Demographic Characteristics of Study Population

The study population comprised individuals from a trachoma endemic region of Southwestern Nepal. Characteristics of the study population are summarized in Table 1. Having TT was significantly associated with older age [O.R. = 1.07 (1.05–1.09), *p*-value = 0.001] and being female [O.R. = 3.1 (2–4.8), *p*-value = 0.001], which is consistent with the literature [3], [26], [27]. The prevalence of *C. trachomatis* infection was 11.3% (22/194) for controls, 48 (27.6%) for those with TF and/or intense trachomatous inflammation (TI), and 16.5% (21/127) for TT cases. Other studies have also found high infection rates among TT cases [23], [28], [29] and high rates among controls [6], [30]–[32].

**Table 1:** Characteristics of Study Population.

Characteristics		Control (n = 232)	TF/TI (n = 204)	TT* (n = 135)
Age in years (mean+/−sd)		30.8 (16.8)	35.2 (18.1)	51 (14.2) †
Sex (%)†	Female (%)	95 (41%)	133 (65.8%)	100 (74.1%)
	Male (%)	137 (59%)	69 (34.2%)	35 (25.9%)
*C. trachomatis*-positive ‡ (%)		22 (11.3%)	48 (27.6%)†	21 (16.5%)

* TT includes cases with or without TF and/or TI.

† Statistically significant difference at α level = 0.001 by two sampled t-test (age) or Pearson chi-square test (sex, *C. trachomatis*-positive).

‡ Amplicor PCR test was used to determine *C. trachomatis* infection status and was available for 194 controls, 174 TF/TI and 127 TT cases.

### Genomic frequencies of 51 inflammatory SNPs among TT cases vs. controls

Because all the cases and controls in the study were of the Tharu ethnic group, we assumed that population admixture would not be a confounder in the analyses. Also, because subjects under age 20 years can have TS and TT [33]–[35], we felt it was reasonable to include them in our study.

An example of the linear array results are shown in Figure 1; all samples yielded appropriate readable bands in the array, suggesting a lack of inhibitors in the PCR and downstream hybridization. Single allele frequencies by TT status were compared with controls for the 51 candidate SNPs, and are summarized in Table 2. We defined major allele as the first allele that appeared in the inflammatory panel, and minor allele as the second allele that appeared in the panel. Genotype frequencies differed significantly by TT compared with controls for TNFA (−308 *G>A*), LTA (252 *G>A*), IL1A (−889 *C>T*), IL1B (F105F), IL6 (−174 *C>G*), VCAM1 (−1594 *T>C*), IL4 (−589 *C>T*), IL4R (Q576R, and I50V), IL9 (T113M), CCR3 (P39L), ADRB2 (Q27E, and T164I), CTLA4 (−318 *C>T*), NOS2A (D346D), and NOS3 (E298D) polymorphisms (Table 3). All observed genotype frequencies between TT cases and controls in our study were in HWE.

**Table 2:** Major Allele Frequencies among 51 SNPs in 36 Inflammatory Genes for Trachomatous Trichiasis (TT) Cases and Controls.

					Major allele frequency		
Gene category	Gene (symbol)	SNP	Nuc change	rs no*	TT (n = 135)	Controls (n = 232)	*P*
Proinflammatory cytokine genes	Tumor necrosis factor- alpha (TNFA)	−308	G⇒A	1800629	0.83	0.68	0.001
	Tumor necrosis factor- alpha (TNFA)	−238	G⇒A	361525	0.97	0.98	0.402
	Lymphotoxin alpha (LTA)	252	G⇒A	909253	0.63	0.49	0.001
	Interleukin 1 alpha (IL1A)	−889	C⇒T	1800587	0.24	0.32	0.023
	Interleukin 1 beta (IL1B) ‡	−1418	C⇒T	16944	0.50	0.42	0.031
	Interleukin 1 beta (IL1B)	F105F	C⇒T	1143634	0.92	0.84	0.001
	Interleukin 6 (IL6)	−572	G⇒C	1800796	0.46	0.45	0.818
	Interleukin 6 (IL6)	−174	C⇒G	1800795	0.95	0.99	0.002
Adhesion molecule genes	Intracelluler adhesion molecule-1 (ICAM1)	K56M	A⇒T	5491	0.89	0.90	0.701
	Intracellular adhesion molecule-1 (ICAM1) ‡	G214R	A⇒G	1799969	0.98	0.99	0.714
	Vascular cell adhesion molecule-1 (VCAM1)	−1594	T⇒ C	1041163	0.79	0.73	0.092
	Selectin E (SELE)	S149R	A⇒C	5361	0.92	0.95	0.510
	Selectin P (SELP)	S330N	A⇒G	6131	0.73	0.80	0.035
	Selectin P (SELP)	V640L	G⇒T	6133	0.98	0.91	0.012
Th1/Th2/Th3 cytokines and related genes	Interleukin-4 (IL4)	−589	C⇒T	2243250	0.47	0.38	0.010
	Interleukin 4 receptor (IL4R)	Q576R	A⇒G	1801275	0.72	0.64	0.034
	Interleukin 4 receptor (IL4R)	S478P	T⇒C	1805015	0.89	0.87	0.723
	Interleukin 4 receptor (IL4R)	I50V	A⇒G	1805010	0.49	0.56	0.078
	Interleukin 5 receptor alpha (IL5RA)	−80	G⇒A	2290608	0.76	0.69	0.034
	Interleukin 9 (IL9)	T113M	C⇒T	2069885	0.96	0.84	0.001
	Interleukin 10 (IL10)	−571	C⇒A	1800872	0.55	0.57	0.817
	Interleukin 13 (IL13)	Intron3	C⇒T	1295686	0.64	0.60	0.272
Chemokines and related genes	Small inducible cytokine subfamily A (a.k.a eotaxin, SCYA11)	A23T	C⇒T	3744508	0.84	0.86	0.666
	Small inducible cytokine subfamily A (a.k.a eotaxin, SCYA11)	−1328	G⇒A	4795895	0.93	0.92	0.773
	Chemokine (C-C motif) receptor 2 (CCR2)	V62I	A⇒G	1799864	0.68	0.69	0.741
	Chemokine (C-C motif) receptor 3 (CCR3) ‡	P39L	C⇒T	5742906	0.99	0.94	0.001
	Chemokine (C-C motif) receptor 5 (CCR5) ‡	32 bp	del	333	0.98	1	0.650
	Chemokine (C-C motif) receptor 5 (CCR5)	−2454	A⇒G	1799987	0.46	0.45	0.746
Miscellaneous	Adrenergic receptor beta 2 (ADRB2)	R16G	A⇒G	1042713	0.47	0.39	0.075
	Adrenergic receptor beta 2 (ADRB2)	Q27E	C⇒G	1042714	0.85	0.77	0.007
	Adrenergic receptor beta 2 (ADRB2) ‡	T164I	C⇒T	1800888	0.99	0.90	0.001
	Fc fragment of IgE, receptor for beta subunit (FCER1B)	E237G	A⇒G	569108	0.79	0.75	0.174
	CD14 antigen (CD 14)	−260	T⇒C	2569190	0.43	0.43	1
	Transforming growth factor-1 beta (TGF1B)	−509	C⇒T	1800469	0.58	0.55	0.488
	Uteroglobin (UGB)	38 G/A	G⇒A	3741240	0.55	0.58	0.354
	T-cell transcription factor 7 (TCF7)	P19T	C⇒A	5742913	0.99	1	0.559
	T-cell transcription factor 7 (TCF7) ‡	UAS	UAS		0.76	0.69	0.034
	Complement component 3 (C3)	R102G	C⇒G	2230199	0.93	0.96	0.140
	Complement component 5 (C5)	I802V	A⇒G	17611	0.60	0.63	0.529
	Colony stimulating factor (CSF2)	I117T	T⇒C	25882	0.52	0.55	0.358
	Cytotoxic T-lymphocyte-associated protein 4 (CTLA4)	−318	C⇒T	5742909	0.85	0.78	0.012
	Cytotoxic T-lymphocyte-associated protein 4 (CTLA4)	T17A	A⇒G	231775	0.60	0.55	0.143
	Leukotriene C4 synthase (LTC4S)	−444	A⇒C	730012	0.90	0.89	0.803
	Nitric oxide synthase-inducible (NOS2A)	D346D	C⇒T	1137933	0.87	0.79	0.005
	Nitric oxide synthase 3-endothelial cell (NOS3)	−948	A⇒G	1800779	0.92	0.89	0.303
	Nitric oxide synthase 3-endothelial cell (NOS3)	E298D	G⇒T	1799983	0.84	0.91	0.006
	Stromal derived factor 1 (SDF1)	3′UTR	3′UTR	1801157	0.82	0.75	0.016
	Vitamin D receptor (VDR)	M1T	C⇒T	2228570	0.33	0.38	0.201
	Vitamin D receptor (VDR)	Intron8	A⇒G	1544410	0.71	0.75	0.261

* The first allele that appeared in the linear array inflammatory panel was referred to as the major allele; the second allele in the panel was referred to as the minor.

† www.ncbi.nlm.nih.gov/SNP.

‡ Total number of controls was 230 for ADRB2 (T164I), ICAM1 (G241R), CCR3 (P39L), CCR5 (WTDEL32), TCF7 (P19T); 231 for IL1B (−1418); total number of TT cases was 134 for TCF7 (UAS).

**Table 3:** Genotype Distribution by TT Status Based on Univariate Analysis.

Gene category	Gene (SNP)	Genotype	TT (n = 135)	Controls (n = 232)	p-value
Proinflammatory cytokine genes	TNFA (−308 G>A)	GG	91 (67%)	105 (45%)	<0.001
		GA	41 (30%)	105 (45%)	
		AA	3 (3%)	22 (10%)	
	LTA (252 G>A)	GG	51 (38%)	55 (24%)	<0.001
		GA	70 (52%)	116 (50%)	
		AA	14 (10%)	61 (26%)	
	IL1A (−889 C>T)	CC	5 (4%)	11 (5%)	0.002
		CT	55 (41%)	127 (55%)	
		TT	75 (56%)	94 (40%)	
	IL1B (F105F)	CC	116 (86%)	160 (69%)	<0.001
		CT	18 (13%)	72 (31%)	
		TT	1 (1%)	0	
	IL6 (−174 C>G)	CC	123 (91%)	227 (98%)	0.02
		CG	11 (8%)	5 (2%)	
		GG	1 (1%)	0	
Adhesion molecule genes	VCAM1 (−1594 T>C)	TT	89 (66%)	118 (51%)	0.001
		TC	35 (26%)	104 (45%)	
		CC	11 (8%)	10 (4%)	
Th1/Th2/Th3 cytokines and related genes	IL4 (−589 C>T)	CC	32 (24%)	25 (11%)	0.004
		CT	64 (47%)	125 (54%)	
		TT	39 (29%)	82 (35%)	
	IL4R (Q576R)	AA	71 (53%)	79 (43%)	<0.001
		AG	53 (39%)	141 (51%)	
		GG	11 (8%)	12 (6%)	
	IL4R (I50V)	AA	37 (28%)	67 (29%)	0.017
		AG	60 (44%)	128 (55%)	
		GG	38 (28%)	37 (16%)	
	IL9 (T113M)	CC	126 (93%)	158 (68%)	<0.001
		CT	9 (7%)	73 (32%)	
		TT	0	1 (0.4%)	
Chemokines and related genes	CCR3* (P39L)	CC	133 (98%)	202 (88%)	0.001
		CT	1 (0.1%)	28 (12%)	
		TT	1 (0.1%)	0	
Miscellaneous	ADRB2 (Q27E)	CC	99 (74%)	134 (58%)	0.011
		CG	33 (24%)	90 (39%)	
		GG	3 (2%)	8 (3%)	
	ADRB2* (T164I	CC	312 (87.1%)	177 (100)	0.001
		CT	45 (12.6%)	0	
		TT	1 (0.3%)	0	
	CTLA4 (−318 C>T)	CC	98 (73%)	134 (58%)	0.016
		CT	34 (25%)	92 (40%)	
		TT	3 (2%)	6 (2%)	
	NOS2A (D346D)	CC	101 (75%)	137 (59%)	0.007
		CT	34 (25%)	93 (40%)	
		TT	0	2 (1%)	
	NOS3 (E298D)	GG	94 (70%)	192 (83%)	0.014
		GT	39 (29%)	38 (16%)	
		TT	2 (1%)	2 (1%)	

* Total number of controls were 230 for ADRB2(T164I), CCR3(P39L), total number of TT cases were same as all (n = 135).

After multivariate logistic analysis controlling for age, sex, TF, TI, and *C. trachomatis* infection status, only certain SNPs remained significantly associated with TT compared to the controls (Table 4). The pro-inflammatory TNFA (−308 *G>A*), and LTA (+252 *G>A*), the adhesion molecule VCAM1 (−1594 *T>C*), and the Th2 cytokine IL-9 (T113M) gene SNPs were significantly associated with TT cases compared with controls. For TNFA (−308 *G>A*), heterozygosity was associated with significantly decreased odds of TT compared with homozygosity for the major genotype [O.R. = 0.45 (0.25–0.81), *p* = 0.008]. Similarly, the odds of TT were less in homozygous minor allele individuals [O.R. = 0.25 (0.09–0.63), *p* = 0.004] compared with individuals with homozygous major allele genotype for the LTA (252 *G>A*) polymorphism.

**Table 4:** Multivariate Analysis of Inflammatory Gene SNPs Associated with TT Cases Compared with Controls.

Gene category	Predicting SNP	Genotype	O.R. (95% C.I.) for TT	*P*
Proinflammatory cytokine genes	TNFA (−308)	GG	Reference	
		GA	0.45 (0.25–0.81)	0.008
		AA	0.19 (0.04–1.08)	0.062
	LTA (252)	GG	Reference	
		GA	0.65 (0.35–1.2)	0.173
		AA	0.25 (0.09–0.63)	0.004
Adhesion molecule genes	VCAM1 (−1594)	TT	Reference	
		TC	0.47 (0.25–0.86)	0.015
		CC	1.06 (0.33–3.4)	0.919
Th1/Th2/Th3 cytokines and related genes	IL9 (T113M)	CC	Reference	
		CT	0.25 (0.10–0.64)	0.004
		TT	Dropped due to colinearity	NA

* Covariates included in multivariable logistic regression were age, sex, TF/TI, and *C. trachomatis* infection status for predicting O.R. s with 95% CI.

Among the SNPs in genes encoding adhesion molecules, TT was associated only with VCAM1 (−1594 *T>C*). The odds ratio for heterozygous individuals was significantly less [O.R. = 0.47 (0.25–0.86), *p* = 0.015] than for individuals with the homozygous major allele genotype (Table 4).

Among the cytokines associated with T helper lymphocyte polarity, we found that the heterozygous IL9 (T113M) genotype was a likely protective factor against TT compared with individuals homozygous for the major genotype (Table 4, O.R. = 0.25 (0.10–0.64), *p* = 0.004).

Although we controlled for age and sex in the analyses as described above, we also stratified subjects by age and sex, controlling for infection, and reanalyzed the data. We found similarly statistically significant results as above (Tables S1, S2, S3, S4).

### Logic regression analysis of interacting SNPs

**Figure 2:**
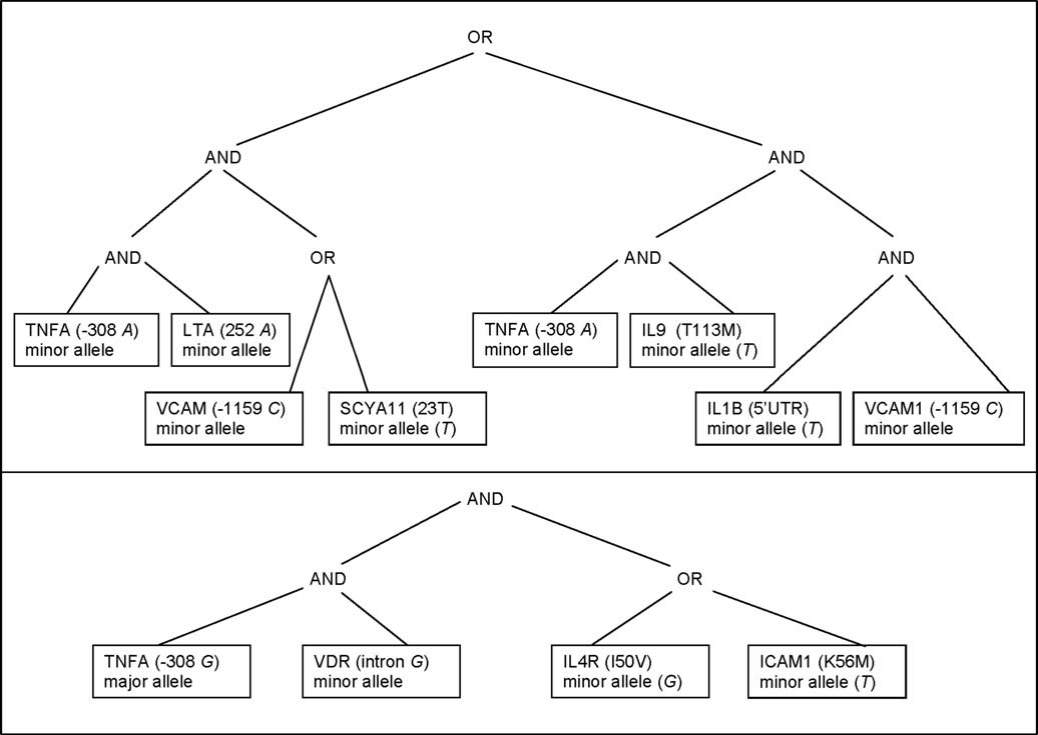
Logic Tree Models for interacting SNPs associated with TT. TOP, L1 is a combination of (1)TNFA(−308 *A*) minor allele and LTA (252 *A*) minor allele and either VCAM-1 (−1594 *C*) minor allele or SCYA 11 (23T) minor allele, or (2) TNFA (−308 *A*) minor allele and IL-9 (113M) minor allele and IL-1B (5′UTR *T*) minor allele and VCAM-1 (−1594 *C*) minor allele. BOTTOM, L2 is a combination of TNFA (−308 *G*) major allele and VDR (intron *G*) minor allele, and either IL-4R (50V) minor allele or ICAM-1 (56M) minor allele: Logit P (TT = 1| risk inflammatory SNPs) = 0.3–1.6*L1+2.6*L2. L1 = ((((TNFA (−308 *A*) minor allele) and (LTA (252 *A*) minor allele)) and ((VCAM1(−1594 *C*) minor allele) and (SCYA 11 (A23T) minor allele))) OR (((TNFA (−308 *A*) minor allele) and (IL-9 (T113M) minor allele)) and ((IL-1B (5′UTR *T*) minor allele) and (VCAM1 (−1594 *C*) minor allele)))). L2 = (((TNFA (−308 *G*) major allele) and (VDR (intron *G*) minor allele)) AND ((IL-4R (I50V) minor allele) or ICAM1 (K56M))).

We further investigated the potential impact of interacting SNPs. Figure 2 shows the logic trees (L) of interacting SNPs that are associated with TT. The logic modeling resulted in Logit P (TT = 1| risk inflammatory SNPs) = 0.3–1.6*L1+2.6*L2. According to logic modeling, TT risk decreased 5 times (e^−1.6^ = 0.2) by the tree (L1) specifying SNP combinations, namely, individuals with a combination of (1) TNFA(−308 *A*) minor allele and LTA (252 *A*) minor allele and either VCAM1 (−1594C) minor allele or SCYA 11 (23T) minor allele, or (2) TNFA (−308 *A*) minor allele and IL-9 (113M) minor allele and IL1B (5′UTR *T*) minor allele and VCAM1 (−1594 *C*) minor allele [odds ratio = 0.2 (95% confidence interval 0.11–0.33), *p* = 0.001, Figure 2A]. In the second tree (L2), TT risk increased 13.5 times [odds ratio = 13.5 (95% confidence interval 3.3–22), p = 0.001] for individuals demonstrating a combination of TNFA (−308 *G*) major allele and VDR (intron *G*) minor allele, and either IL4R (50V) minor allele or ICAM1 (56M) minor allele (Figure 2B).

## Discussion

The pathogenesis of trachoma, as in many diseases, is related to the complex interplay of the environment, pathogen, epigenetic factors and the host immune response that is intimately related to host genetic susceptibility to disease. Susceptibility to cerebral malaria [36], tuberculosis [37], HIV [38], rheumatoid arthritis [39], multiple sclerosis [40], and Alzheimer disease [41] are among the diseases found to be associated with SNPs in inflammatory genes. To date, there are minimal studies linking polymorphisms in inflammatory genes with the severe end-stage disease of trachoma, TT.

In this study, we focused on TT as a more accurate clinical sign of disease outcome than active disease (TF and/or TI) and, therefore, less susceptible to misclassification and more suitable for genetic studies of susceptibility. For TF and TI, it is not clear which individuals with inflammation will go on to develop sequela or clear the inflammation without progression to disease. Thus, we considered it best not to classify these individuals as cases or controls. TF and TI were controlled for in multivariate logistic regression analysis for these reasons. We also controlled for *C. trachomatis* infection because other infectious diseases such as malaria, tuberculosis and HIV-1 have been shown to be independently associated with SNPs [36]–[38], although the association of specific SNPs with chlamydiae has not been evaluated.

We found an association between TT and polymorphisms in two genes that are members of the TNF superfamily: TNFA (−308 G>) and LTA (intron *G>A*). Both genes are located on chromosome 6, as is the major histocompatibility complex (MHC), and are highly similar structurally and functionally [42]. The cytokines encoded by these two genes bind the same receptor, which is upregulated by IFN-γ [43]. TNF-α is a major proinflammatory cytokine secreted by an array of leucocytes in chlamydial infected tissues [44]. It was first shown *in vitro* that human recombinant TNF-α inhibited the growth of *C. trachomatis* [45]. Following this finding, the cytotoxic effect of TNF-α on *C. trachomatis* infected cells was shown in a murine model of chlamydial genital tract infections [46]. TNF-α mRNA transcripts and protein levels have been reported to be significantly higher in subjects who were infected with chlamydiae than those who were not infected [11], [13], [14]. Conjunctival TNF-α mRNA levels have also been found to be positively correlated with duration of infection [13].

The TNFA (−308) polymorphism, located in the promoter region, has been extensively studied for genetic association with a variety of diseases. We found −308 *GA* (heterozygous) and *AA* genotypes to be associated with a decreased odds of TT whereas a previous study found an association with susceptibility to TT [20]. This might be due to geographic and population differences. However, it is not clear if there was any adjustment for infection or active disease status, as in our study. In addition, the p-value cut-off for statistical significance was 0.048, which is not conservative for a population based genetic association study. In the same study, they also found that the TNFA (−308) *GA* genotype was correlated with increased TNF-α protein production [20]. However, this finding depended on only three subjects with the *AA* genotype. Their p-value by ANOVA was only 0.043, and by Kruskall-Wallis was 0.075.

Elevated TNF-α levels have been considered protective for *C. trachomatis* infection and essential for resolution of active disease but, it has also been associated with chronic disease [11], [13]. More importantly, the effect of TNFA (−308 *G*) carried an increased risk of TT if an individual also carried the VDR (intron *G*), IL-4R (50V), and ICAM-1 (56M) minor allele. However, further quantitative protein studies will be required to characterize the role of this protein and SNP in TT.

The LTA gene, also known as TNFB, is primarily expressed in lymphocytes and has been associated with regulating CD8+ T lymphocytes. Several animal models of genital chlamydial infection have shown that CD8+ T cells are protective against chlamydial infection and are necessary for infection clearance [47], [48]. In TT cases, elevated CD8+ T lymphocytes have been identified in conjunctival samples [49], [50]. We found that the LTA (252 *A*) variant was significantly associated with a decreased odds of TT. Interestingly, Natividad et.al. [20] found an association between LTA (252 *G*) and TT. We found that the *A* allele decreased the odds of TT, given that *GG* was our reference while *AA* homozygosity was their reference.

One of the major factors involved in the migration of leukocytes to the foci of infection are a family of adhesion molecules. VCAM-1 is a member of the IgG superfamily that is expressed on endothelial cells and leads to the adhesion of lymphocytes via α4β1 integrins [51]. In peripheral endothelia, especially in the conjunctiva, polymorphonuclear leukocytes act as generators of the inflammation processes [52], [53]. To our knowledge, we are the first to report an association between TT and the VCAM1 (−1594 *TC*) polymorphism where heterozygosity (*TC*) decreased the odds of TT [O.R. = 0.47 (0.25–0.86), *p*-value = 0.015-MLR]. ICAM-1 and VCAM-1 have been found to be significantly elevated in the vasculature after genital chlamydial infection in the murine model [54]–[56]. VCAM-1 has also been associated with a Th1 immune response and clearance of chlamydial infection [55]. Interestingly, VCAM-1 is induced by LTA, and by the LTA (252 *A*) SNP [57]. Thus, variation in LTA and adhesion molecules may increase homing of T cells to the mucosa, which might play a significant role in clearance and prevention of disease progression to TT.

It is well-documented that cellular-mediated immunity is critical in eliciting protection against *C. trachomatis* [58]–[60]. We were therefore interested in studying multiple SNP polymorphisms within the Th1/Th2/Th3 cytokine family. In our previous study, we demonstrated that Th1 and some Th2 cytokines were protective against chronic, scarring trachoma [14]. The IL9 gene loci is assigned to chromosome 5q31.1, similar to other Th2 cytokines, including IL-4, IL-5 and IL-13 [61]. On the IL-9 protein, a cytosine at amino acid position 338 in exon 5 is substituted by thymine, resulting in a non-synonymous mutation from a hydrophilic threonine to a hydrophobic methionine at position 113 (T113M). In our study, the IL-9 major allele frequency (T113M) differed significantly between cases (0.96) and controls (0.84, *p* = 0.001, Table 2), and heterozygosity exhibited protection against TT in our population (O.R. = 0.25, *p* = 0.004, Table 4). IL-9 is a pleiotropic cytokine produced primarily by Th2 lymphocytes [62]. It has been associated with proliferation and maturation of both lymphoid and myeloid cell progenitors [63] as well as growth factor for activated T lymphocytes [64]. Physiologically, it causes decreased levels of the proinflammatory cytokines TNF-α, IL-12p40, and IFN-γ while increasing the levels of anti-inflammatory IL-10 [65], [66]. The association of elevated IL-10 mRNA expression and protein levels with TS and TT has been well documented [12]–[14], [67], suggesting a plausible biological role for the IL9 polymorphism in disease pathogenesis.

Logic regression is particularly appropriate for determining multiple interactions among many polymorphisms, and accounts for multiple comparisons. Our results indicate the importance of the effect of interacting gene variants on disease susceptibility. We showed that different combinations of SNPs had a synergistic effect on TT. The combination of TNFA (−308 *G*) major allele, and VDR (intron *G*) minor allele, and either IL4R (I50V) minor allele or ICAM1 (K56M) minor allele significantly increased the odds of TT [odds ratio = 13.5 (95% confidence interval 3.3–22), p = 0.001]. Individually, none of these SNPs was statistically associated with TT. SNP interactions that are repeatedly detected might indicate immune pathways that could be contributors to disease pathogenesis, and could be used as markers for individuals at risk for disease progression.

We confirmed significant associations between TNFA and LTA polymorphisms and TT, and showed for the first time an association between VCAM1 (−1594 *TC*) and IL9 (T113M) polymorphisms and protection from TT. More importantly, this study indicates that interacting SNPs likely have a synergistic effect on disease. Our findings lay the foundation for selecting additional genes to study that may be in linkage disequilibrium with the SNPs that we found to be associated with TT. In addition, it will now be possible to determine whether dysregulated cytokine and/or chemokine production are associated with the susceptibility variants. These will be important next steps in trachoma research.

## Supporting Information

Table S1 Characteristics of Matched Population
(0.09 MB DOC)

Table S2 Major Allele Frequencies among 51 SNPs in 36 Inflammatory Genes for Trachomatous Trichiasis (TT) Cases and Controls
(0.04 MB DOC)

Table S3 Genotype Distribution by TT Status Based on Univariate Analysis
(0.05 MB DOC)

Table S4 Multivariate Analysis of Inflammatory Gene SNPs Associated with TT Cases Compared with Controls
(0.05 MB DOC)
