# Supplementary material for: Identification of Novel Single Nucleotide Polymorphisms in Inflammatory Genes as Risk Factors Associated with Trachomatous Trichiasis
Source: PLoS One. 2008 Oct 31;3(10):e3600. doi: 10.1371/journal.pone.0003600 (PMC2572999; doi:10.1371/journal.pone.0003600)
Supplement: Table S1 — Characteristics of Matched Population (0.09 MB DOC) [file pone.0003600.s001.doc]

Table S1. Characteristics of Matched Population

| Characteristics |  | Control (n=82) | TF/TI (n=49) | TT (n=82) |
| --- | --- | --- | --- | --- |
| Age in years (mean +/- sd) |  | 46.02 (12.24) | 43.38 (12.46) | 46.02 (12.24) |
| Sex (%) | Female (%) | 42 (51.2%) | 33 (32.6%) | 53 (64.6%) |
|  | Male (%) | 40 (48.8%) | 16 (67.4%) | 29 (35.4%) |
| *C. trachomatis*-positive (%) |  | 3 (3.8%) | 10 (21.7%)^†^ | 13(16.5%)* |

*p=0.009

^†^ p=0.002
