# Supplementary material for: Identification of Novel Single Nucleotide Polymorphisms in Inflammatory Genes as Risk Factors Associated with Trachomatous Trichiasis
Source: PLoS One. 2008 Oct 31;3(10):e3600. doi: 10.1371/journal.pone.0003600 (PMC2572999; doi:10.1371/journal.pone.0003600)
Supplement: Table S2 — Major Allele Frequencies among 51 SNPs in 36 Inflammatory Genes for Trachomatous Trichiasis (TT) Cases and Controls (0.04 MB DOC) [file pone.0003600.s002.doc]

Table S2. Major Allele Frequencies among 51 SNPs in 36 Inflammatory Genes for Trachomatous Trichiasis (TT) Cases and Controls

|  |  |  |  |  | Major allele frequency | |  |
| --- | --- | --- | --- | --- | --- | --- | --- |
| Gene category | Gene (symbol) | SNP | Nuc change | rs no* | controls (n=82) | TT (n=82) | P |
| Proinflammatory cytokine genes | Tumor necrosis factor- alpha (TNF-α) | -308 | G⇒A | 1800629 | 0.66 | 0.81 | 0.007 |
|  | Lymphotoxin alpha (LTA) | 252 | G⇒A | 909253 | 0.70 | 0.89 | 0.003 |
| Adhesion molecule genes | Intracelluler adhesion molecule-1 (ICAM1) | K56M | A⇒T | 5491 | 0.93 | 0.85 | 0.026 |
| Th1/Th2/Th3 cytokines and related genes | Interleukin-9 (IL-9) | T113M | C⇒T | 2069885 | 0.89 | 0.96 | 0.018 |
| Miscellaneous | Stromal derived factor 1 (SDF1) | 3’UTR | 3’UTR | 1801157 | 0.76 | 0.86 | 0.034 |
