# Supplementary material for: Identification of Novel Single Nucleotide Polymorphisms in Inflammatory Genes as Risk Factors Associated with Trachomatous Trichiasis
Source: PLoS One. 2008 Oct 31;3(10):e3600. doi: 10.1371/journal.pone.0003600 (PMC2572999; doi:10.1371/journal.pone.0003600)
Supplement: Table S3 — Genotype Distribution by TT Status Based on Univariate Analysis (0.05 MB DOC) [file pone.0003600.s003.doc]

Table S3. Genotype Distribution by TT Status Based on Univariate Analysis.

| Gene category | Gene (SNP) | Genotype | TT (n=82) | Controls (n=82) | p-value |
| --- | --- | --- | --- | --- | --- |
| Proinflammatory cytokine genes | TNF-α (-308 G>A) | GG | 53 (64.6%) | 34 (41.5%) | 0.007 |
|  |  | GA | 27 (33%) | 41 (50%) |  |
|  |  | AA | 2 (2.6%) | 7 (8.5%) |  |
|  | LTA (252 G>A) | GG | 32(39%) | 18 (22%) | 0.005 |
|  |  | GA | 41 (50%) | 40 (48.8%) |  |
|  |  | AA | 9 (11%) | 24 (29.2%) |  |
| Adhesion molecule genes | ICAM (K56M) |  | 60 (73.2%) | 73 (89%) | 0.029 |
|  |  |  | 21 (25.6%) | 8 (9.8%) |  |
|  |  |  | 1 (1.2%) | 1 (1.2%) |  |
| Th1/Th2/Th3 cytokines and related genes | IL-9 (T113M) | CC | 76 (92.7%) | 64 (78%) | 0.008 |
|  |  | CT | 6 (7.3%) | 18 (22%) |  |
|  |  | TT | 0 | 0 |  |
