# Supplementary material for: Identification of Novel Single Nucleotide Polymorphisms in Inflammatory Genes as Risk Factors Associated with Trachomatous Trichiasis
Source: PLoS One. 2008 Oct 31;3(10):e3600. doi: 10.1371/journal.pone.0003600 (PMC2572999; doi:10.1371/journal.pone.0003600)
Supplement: Table S4 — Multivariate Analysis of Inflammatory Gene SNPs Associated with TT Cases Compared with Controls (0.05 MB DOC) [file pone.0003600.s004.doc]

Table S4. Multivariate Analysis of Inflammatory Gene SNPs Associated with TT Cases Compared with Controls

| Gene category | Predicting SNP | Genotype | O.R. (95% C.I.) for TT | *P* |
| --- | --- | --- | --- | --- |
| Proinflammatory cytokine genes | TNF-α (-308) | GG | Reference |  |
|  |  | GA | 0.49 (0.25-0.97) | 0.041 |
|  |  | AA | 0.14 (0.02-1.3) | 0.08 |
|  | LTA (252) | GG | Reference |  |
|  |  | GA | 0.57 (0.27-1.2) | 0.156 |
|  |  | AA | 0.23 (0.08-0.65) | 0.006 |
| Th1/Th2/Th3 cytokines and related genes | IL-9 (T113M) | CC | Reference |  |
|  |  | CT | 0.29 (0.10-0.84) | 0.01 |
|  |  | TT | Dropped due to colinearity | NA |
